# Channel morphology effect on water transport through graphene bilayers

**DOI:** 10.1038/srep38583

**Published:** 2016-12-08

**Authors:** Bo Liu, Renbing Wu, Adrian Wing-Keung Law, Xi-Qiao Feng, Lichun Bai, Kun Zhou

**Affiliations:** 1 Environmental Process Modelling Centre, Nanyang Environment and Water Research Institute, Nanyang Technological University, 50 Nanyang Avenue, Singapore 639798, Singapore; 2 School of Mechanical and Aerospace Engineering, Nanyang Technological University, 50 Nanyang Avenue, Singapore 639798, Singapore; 3 School of Civil and Environmental Engineering, Nanyang Technological University, 50 Nanyang Avenue, Singapore 639798, Singapore; 4 Department of Engineering Mechanics, Tsinghua University, Beijing 100084, China

## Abstract

The application of few-layered graphene-derived functional thin films for molecular filtration and separation has recently attracted intensive interests. In practice, the morphology of the nanochannel formed by the graphene (GE) layers is not ideally flat and can be affected by various factors. This work investigates the effect of channel morphology on the water transport behaviors through the GE bilayers via molecular dynamics simulations. The simulation results show that the water flow velocity and transport resistance highly depend on the curvature of the graphene layers, particularly when they are curved in non-synergic patterns. To understand the channel morphology effect, the distributions of water density, dipole moment orientation and hydrogen bonds inside the channel are investigated, and the potential energy surface with different distances to the basal GE layer is analyzed. It shows that the channel morphology significantly changes the distribution of the water molecules and their orientation and interaction inside the channel. The energy barrier for water molecules transport through the channel also significantly depends on the channel morphology.

Recently, the application of multi-layered graphene-derived functional thin films for molecular filtration and separation has attracted intensive interests due to their unique lamellar porous microstructures^1^,^2^,^3^,^4^,^5^,^6^,^7^,^8^,^9^. Different from the single layer graphene (GE) with elaborately created nanopores, in the layered GE-derived thin films, molecules with appropriate sizes permeate through the interconnected nanochannels formed between adjacent GE layers^6^,^7^,^10^. Compared with the nanopores in the single layer GE, these nanochannels allow fast molecule transport and their sizes can also be efficiently controlled for flexible filtration tunability^7^,^10^. One recent experimental work also demonstrated that the multiple-layer GE can be used to create nanoscale capillaries with atomic-scale precision for efficient molecular transport^11^. Hence, the multilayer GE-derived thin films are becoming more promising than the porous single layer GE nanosheet in novel molecule filtration applications, particularly for seawater desalination.

One important target during the fabrication of desalination thin films is to achieve fast water permeation while retain high filtration rate. Previous studies have demonstrated that when confined in nanochannels with sizes ranging from sub-nanometer to several nanometers, fluids can exercise unconventional and sometimes even counterintuitive behaviors^12^,^13^,^14^,^15^,^16^,^17^. Such behaviors are mainly due to the fact that the interactions among the fluid ions or molecules change dramatically when they are in close contact with the confining walls^15^,^18^. Both molecular simulations and experimental works have revealed that the flow rate of water through a few layered GE is orders of magnitude higher than that predicted by the conventional Hagen-Poiseuille law^7^,^14^,^19^. This high transport rate has been attributed to the inherent smoothness of the interior wall of the GE layers and the high capillary driving pressure induced by the narrow nanochannels^7^,^14^.

In most molecular simulations or theoretical analyses, the GE layers were treated as ideally smooth and flat, and the distance between the adjacent GE layers was assumed to be constant along the nanochannel length direction^7^,^20^,^21^. However, in practice, the morphology of the nanochannel formed by the GE layers is usually corrugated and affected by various factors. Previous experimental studies have demonstrated that when the chemically converted GE nanosheets are immerged into water, the corrugation of the GE layers can be readily controlled at the nanometer scale by hydrothermal treatment^22^. Such controllable corrugation is advantageous for attaining tunable nanofiltration performance of the GE-based membranes. Meanwhile, during the fabrication of GE thin films by vacuum filtration or spin coating from GE oxide solution, small GE flakes can fold and lie between two other large flakes^7^. In this scenario, the nanochannel becomes asperous. In addition, nanoparticles such as carbon nanotubes are sometimes intentionally intercalated inside the nanochannel to modify the nanochannel morphology for the increase of water permeability^19^. In practical applications, GE thin films need to be coated on certain substrate for supporting. The surface roughness of the substrate would also have great impact on the morphology of the nanochannel. With the change of the morphology of the nanochannel, the water transport behaviors inside it might deviate significantly from those observed in ideally flat GE layers due to the change of the interfacial interaction between the water molecules and the confining walls.

Using molecular dynamics (MD) simulations, Mittal and Hummer studied the interfacial features of water molecules confined between two planar surfaces, and found the liquid-solid interfacial free energy changes dramatically as the surface morphology becomes rough^23^. Jabbarzadeh *et al*. conducted MD simulations to investigate the transport behaviors of hexadecane flow between two sinusoidal atomic planes, and found that the relative size of the fluid molecules and the wall morphology determine the slip or nonslip boundary conditions^13^. Xu *et al*. studied the effect of wall roughness on fluid transport resistance in carbon nanotubes and demonstrated that both the transport resistance and nominal viscosity of the fluid increase as the roughness increases^23^. A recent experimental study also showed that the water permeation rate inside the carbon nanotubes highly depends on their radius^24^. All these studies indicate that the morphology of the liquid-solid interaction interface plays a critical role in determining the liquid transport behaviors.

To understand how the fluid permeation through a few layered GE thin films is affected by the morphology of the nanochannels, this study investigates the water transport behaviors through the nanochannel formed inside the GE bilayers with curving shapes via MD simulations. The effects of channel morphology are examined by exploring the water flow velocity, transport resistance, density distribution, water dipole moment orientation, hydrogen bonds and the potential energy surface. The results from the present study can not only provide guidance for novel engineering membrane design, but also contribute to promote the application of GE-based thin films in future water desalination and purification technologies.

## Results and Discussions

### Water flow velocity

**Figure 1:**
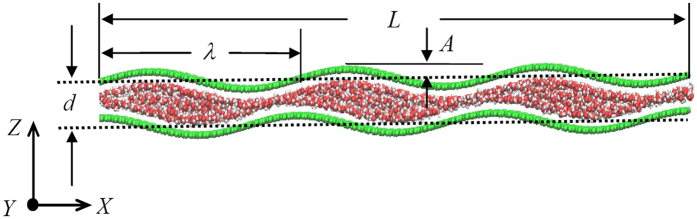
Schematic of the atomic configuration of the GE bilayer. Two GE nanosheets (green color) are deformed along the channel thickness z-direction in a sinusoidal wave profile and separated by a distance of *d*. Water molecules (red and white colors) are filled inside the nanochannel between the GE layers and driven to flow inside. The dash lines indicate the mid-planes of the two GE layers.

Two GE nanosheets with a curving morphology along the thickness direction (*z*-direction) is separated by an interlayer distance *d* to form the nanochannel for water molecule transport, as shown in Fig. 1. The lattice constant of GE layers is set to be 1.42 Å. The morphology profile of the lower and upper GE layers are described respectively by two sinusoidal wave functions:


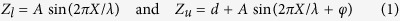


where *A* is the wave amplitude, *X* is the atomic position along the longitude *x*-direction, *λ* is the wave length, and *φ* accounts for the phase angle difference between the two sinusoidal profiles. It is noted that the interlayer distance *d* is defined as the distance between the mid-planes of the two GE layers. For a non-zero phase angle *φ*, the two GE layers corrugate in a non-synergic pattern.

**Figure 2:**
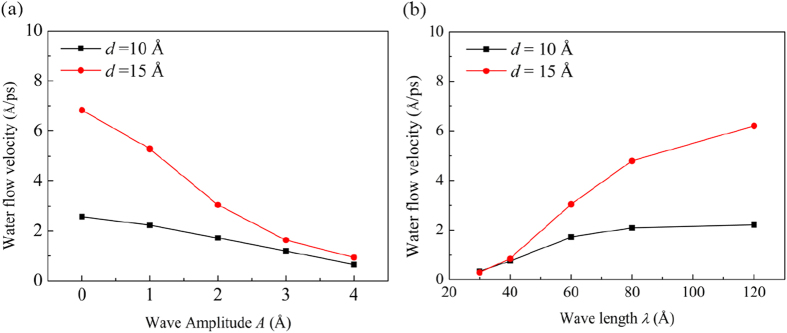
Dependence of water flow velocity *v* along the *x*-direction on (**a**) the wave amplitude *A* and (**b**) the wave length *λ* for different interlayer distances *d* = 10 and *d* = 15 Å. In (**a**), *λ* is fixed at 60 Å, and in (**b**), *A* is fixed at 2 Å.

The steady water flow velocity *v* along the longitude *x*-direction at various wave amplitudes *A* and wave lengths *λ* for both the interlayer distances *d* = 10 and *d* = 15 Å are presented in Fig. 2. Here, the steady *v* is obtained by averaging the *x*-component velocity of all the water molecules inside the channel over a period of 100 ps at the end of the simulation. The phase difference angle *φ* is set as zero at the moment.

It is found that as *A* increases or *λ* decreases, *v* decreases monotonically. In addition, for a larger *d*, *v* is always larger for any given *A* or *λ*. Moreover, *v* becomes more sensitive to the change of channel morphology when the interlayer distance is larger. As *A* increases from 0 to 4 Å, *v* decreases by 75% and 86% for *d* = 10 and *d* = 15 Å, respectively. When *λ* decreases from 120 to 30 Å, *v* decreases by 85% and 95% for or *d* = 10 and *d* = 15 Å, respectively. The water flow has an increased sensitivity to the channel morphology when the interlayer distance increases, an opposite phenomenon compared with a macroscale flow. This is because when the water molecules flow in the GE bilayer, they are severely confined by the GE layers and tend to form a layered structure^25^. At a small interlayer distance of 10 Å, the water molecules form a clear layered structure. When the interlayer distance increases to 15 Å, the confinement of the GE layers on the water molecules weakens and the layered-structure becomes less distinguishable and gets close to the structure of the bulk water. The weakening of the confinement by the GE layers and the change of the distribution structure of the water molecules may account for the higher sensitivity of flow velocity on the channel morphology for a larger interlayer distance of 15 Å.

**Figure 3:**
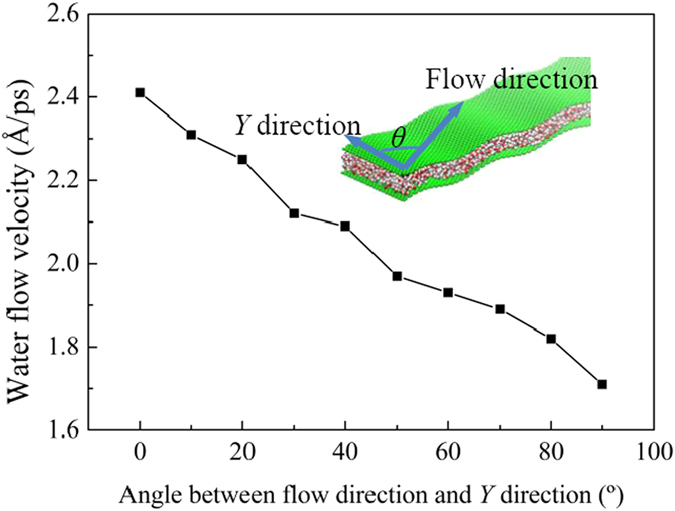
Dependence of the water flow velocity on the water flow direction for *λ* = 60, *A* = 2 and *d* = 10 Å.

It should be noted that in real applications, when the water molecules are forced to permeate a GE thin film, they may flow in arbitrary directions inside the nanochannels of the film, depending on the detailed surface structure and morphology of the channel. In the present model, the water flow direction is forced to be along the *x*-direction, but it can be tuned through changing the direction of the driving force applied. Figure 3 shows the dependence of the water flow velocity on the flow direction for the case of *λ* = 60, *A* = 2 and *d* = 10 Å. As the angle *θ* between the water flow direction and the *y-*direction increases, the water flow velocity decreases monotonically. For *θ* = 0°, the water flow direction is along the *y*-direction and perpendicular to the wave length direction of the sinusoidal GE layers, and the flow velocity is the highest. In this case, the curvature of the GE layers does not block the movement of the water molecules and they flow across the nanochannel as similar to flow between two flat GE layers. Although, the simulation results indicate that the water flow velocity is still influenced by the change of the GE layer morphology even when *θ* = 0°.

**Figure 4:**
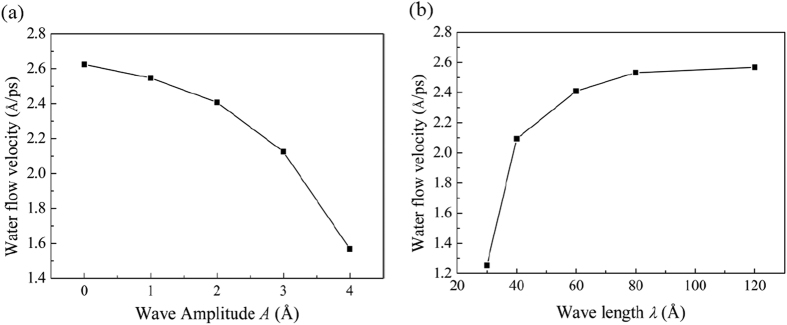
Dependence of water flow velocity *v* for *θ* = 0° (flow along the *y*-direction) on (**a**) wave amplitude *A* and (**b**) wave length *λ* for the interlayer distance *d* = 10 Å. In (**a**), *λ* is fixed at 60 Å, and in (**b**), *A* is fixed at 2 Å.

Figure 4 presents the dependence of water flow velocity *v* along the *y*-direction on the wave amplitudes *A* and wave lengths *λ* for *d* = 10 Å. Similar to the case when *θ* = 90° (water flows along the *x*-direction), *v* for *θ* = 0° also decreases monotonically with the increase of *A* or the decrease of *λ*. However, the dependence of *v* for *θ* = 0° is weaker than that for *θ* = 90°. As the amplitude *A* increases from 0 to 4 Å, for *θ* = 0°, *v* deceases less than 50% which is much smaller than that of 75% for *θ* = 90°.

In the above discussion, the phase angle difference *φ* between the upper and lower GE layers is taken as zero. With *φ* = 0°, the upper and lower GE layers corrugate at the same pace and the distance between them remains constant along the *x*-direction. However, in real GE thin films, the distance between two adjacent GE layers varies at different locations. When *φ* ≠ 0°, the distance Δ*Z* between the upper and lower GE layers depends on *φ* and can be written as


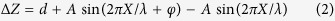


The first order differentiation of Δ*Z* with respect to *X* gives


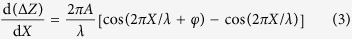


Let 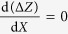, one obtains 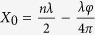
with *n* being an positive integral number. The substitution of *X*_0_ into Eq. 2 with *n* taking an odd number gives the minimum distance between the GE layers as


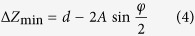


For an even *n*, one obtains the maximum distance as


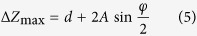


**Figure 5:**
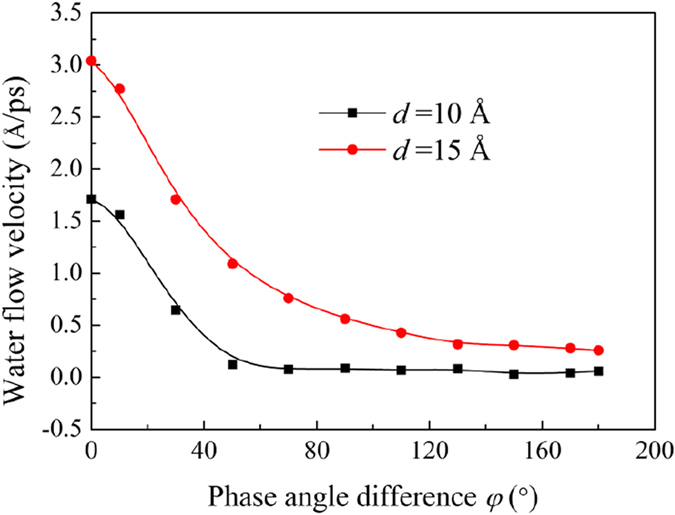
Water flow velocities along the *x*-direction at different phase angle difference *φ* for *d*** = 10 and** *d* = 15 Å. The wave amplitude and wave length are set as *A* = 2, *λ* = 60 Å.

The previous study has demonstrated that the water flow rate inside the flat GE bilayer highly depends on the distance between the GE layers, and increases as the distance increases^25^. Hence, it can be predicted from Eqs. 4 and 5 that the water flow velocity *v* highly depends on the phase angle *φ*. Figure 5 shows the simulation results of the variation of *v* along the *x*-direction as the phase angle difference *φ* increases from 0° to 180° for both the interlayer distances *d* = 10 and *d* = 15 Å. It is found that as *φ* increases, *v* decreases monotonically, indicating that *v* is mostly determined by the minimum distance Δ*Z*_min_. Interestingly, *v* decreases significantly when 0° < *φ* < 70°. When *φ* > 70°, *v* stays almost unchanged for the case of *d* = 10 Å, and decreases slightly for *d* = 15 Å.

For a non-zero phase angle between the upper and lower GE layers, the water molecules inside the GE bilayers behave similar to a lubrication film between two incommensurate surfaces in contact. The Frenkel–Kontorova model^26^ was previously adopted to describe the friction between such lubrication films and the confining surfaces^27^. In this model, the lubrication film is described as a chain of harmonically interacting particles with a periodic lattice structure subjected to an external periodic sinusoidal potential imposed by the supporting substrate of these particles. It has been demonstrated that the interaction strength between the particles would dramatically affect the friction force between the lubrication film and the substrate^28^. Moreover, the incommensurate ratio among the lattice constant of the particles and the periodic lengths of the two substrate potentials also play significant role in affecting the friction behaviors of the lubrication film^27^.

For the water molecules transport across the GE bilayers with a non-zero phase angle difference, the flow velocity is determined by the minimum distance between the upper and lower GE layers. As the phase angle difference increases, the minimum distance decreases, leading to the changes of the distribution and thus the interaction strength of the water molecules. Hence, the friction between the water molecules and the GE layers changes accordingly, leading to the decrease of the flow velocity. Besides introducing a phase angle difference to the two GE layers, the incommensurate between them may also be considered by using different periodic lengths, wave amplitudes, and C-O interaction strengths. Further studies are needed to fully understand the water transport behaviors inside the GE bilayers with incommensurate.

### Water transport resistance

The flow rate of a pressure-driven fluid inside a nanochannel is closely correlated with the transport resistance which can be quantified by three quantities: the deceleration *a* of the fluid when it transports freely inside the nanochannel, the effective shear force exerted by the confining wall and the viscosity of the fluid. These three quantities are closely correlated^1^,^14^,^29^. The effective shear force can be estimated as *τ* = *Nma*/*S*, where *N* is the total number of water molecules inside the channel, *m* the atomic mass of a water molecule, and *S* the contacting area between water and the GE wall. The nominal viscosity can also be calculated as *η* = *τ*/6*vw*. Due to the curvature of the GE layers, the contacting area needed for the calculation of the shear force *τ* and the viscosity *η* is difficult to be well defined. Hence, in this study, the deceleration *a* is adopted as the parameter to demonstrate the overall effective resistance imposed on the water molecules as they flow through the asperous nanochannels formed by the GE layers.

**Figure 6:**
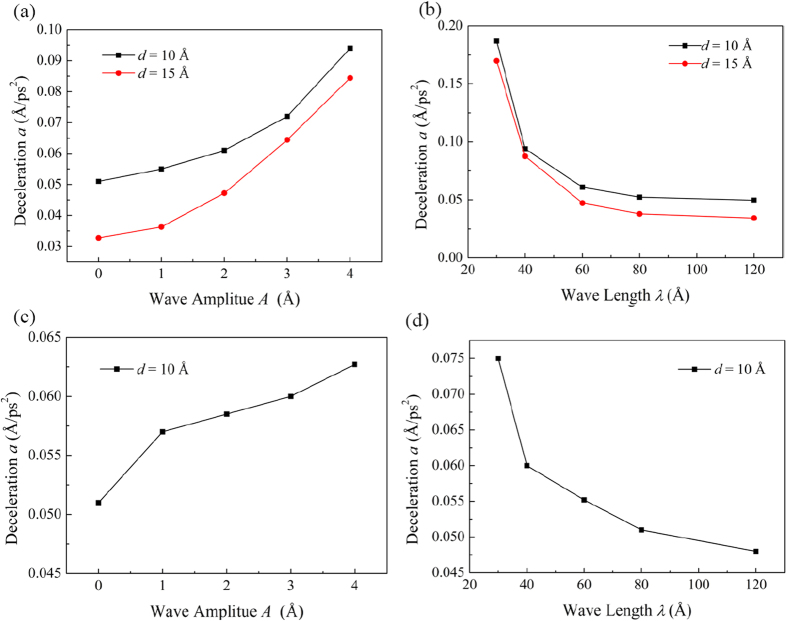
Water deceleration *a* along the (**a,b**) *x*-direction (*θ* = 0°) and (**c,d**) *y*-direction (*θ* = 90°) for different wave amplitudes *A* and wave lengths *λ*. In (**a**) and (**c**), *λ* is fixed at 60 Å. In (**b**) and (**d**), *A* is fixed at 2 Å.

To calculate the decelerations for different channel morphologies, separate simulations have been conducted. During the simulation, the water molecules are first accelerated to the velocity of 2.5 Å/ps in a period of 10 ps by imposing a driving force with an appropriate magnitude. Afterwards, the driving force is removed and the simulation is conducted for 30 ps. During every 0.1 ps, the average velocity of the water molecules along the water flow direction is recorded for the calculation of *a*. Both the decelerations of the water flow along the *x*- and *y*-directions for different channel morphologies are shown in Fig. 6.

It is observed that for a larger interlayer distance *d*, the deceleration *a* is always smaller at any given wave amplitude *A* or wave length *λ*, but the difference becomes smaller when *A* increases and *λ* decreases. In addition, *a* increases with the increase of *A* or with the decrease of *λ* for both the water flows along the *x*- and *y*-directions. However, when the water flows along the *y*-direction (*θ* = 0°), the effects of *A* and *λ* on the deceleration *a* are less prominent than those for the *x*-direction (*θ* = 90°). The dependences of *a* on both *A* and *λ* show close correlation with those of the water velocity *v* (Figs 2 and 4). A larger *a* would always correspond to a smaller water flow velocity *v*, and a larger increase of *a* due to the change of the channel morphology also corresponds to a more intensive decrease of *v*.

An important phenomenon worth noting is that when *A* < 2 Å and *λ* > 60 Å, the deceleration *a* for water flowing along the *x*-direction is close to that for the *y*-direction. The water flow velocities for these two flow directions are also close to each other (Figs 2 and 4). However, for a large *A* > 2 Å or a small *λ* < 60 Å, the deceleration *a* (velocity *v*) for water flowing along the *y*-direction becomes much smaller (larger) than that for the *x*-direction. This result indicates that the blocking effect of the GE walls for water flow along the *x*-direction becomes significant only when *A* is large or *λ* is small.

**Figure 7:**
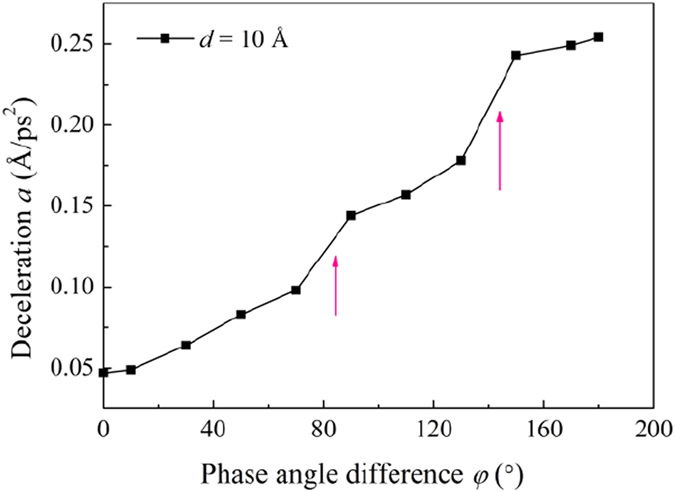
Water flow deceleration *a* along the *x*-direction at different phase angle difference *φ* for *d* = 10 Å. The wave amplitude and wave length are set as *A* = 2, *λ* = 60 Å.

The deceleration *a* at different phase angle difference *φ* has also been calculated, as shown in Fig. 7. As *φ* increases, *a* increases monotonically. Interestingly, at *φ* = 70° and *φ* = 130°, there are two jumps for *a*. These jumps are closely related to the change of the water structure as the minimum distance between the upper and lower GE layers decreases with the increase of *φ* (Eq. 4). The detailed water structure change will be explained further in the discussions below.

### Water molecule velocity profiles

**Figure 8:**
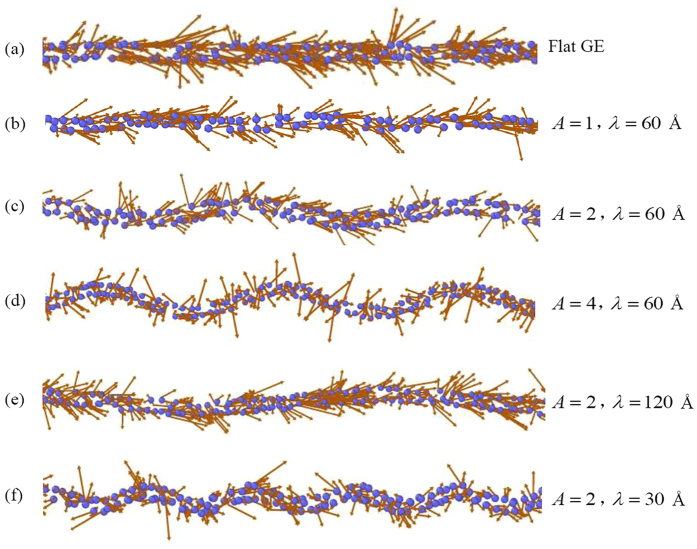
(**a–e**) Representative flow velocity profiles of the water molecules inside the GE bilayer nanochannel with different morphology parameters for the case of *d* = 10 Å. For clarity, one water molecule is represented by one blue point and the arrow shows its velocity direction.

Intuitively, as the confining GE layers become asperous, they extrude into the water body inside the channel and serve as “stumbling blocks” for the transport of water molecules, perturbing their uniform movement and increasing the transport resistance. Several representative snapshots of the velocity profile of the water molecules are shown in Fig. 8 to provide understanding of the water transport behaviors from a molecular point of view. It can be observed that for the wave amplitude *A* = 0 Å, i.e., when the GE layers are flat (Fig. 8(a)), the water molecules move uniformly along the positive *x*-direction though some of them have velocity components along the channel thickness (*y*) direction. The non-zero velocity along the *y*-direction is consistent with the observation of the non-zero radial velocity in previous works which investigate the water transport through carbon nanotubes^30^,^31^. Such non-zero velocity may be caused by the thermal vibration of the water molecules and their strong interaction with the confining GE layers.

When the GE layers become asperous, for a small wave amplitude *A* = 1 Å (Fig. 8(b)), the flow directions of the water molecules retain uniform and not much change occurs as compared to that for flat GE layers. However, as *A* increases, the water movement along the *x*-direction is blocked by the GE layers and the flow directions of the water molecules diverge significantly. Such blocking effect can be evidenced by the observation that more water molecules tend to have velocity directions normal (instead of tangential) to the confining wall surfaces and some water molecules even have negative velocity component along the *x*-direction, indicating the existence of local back flows.

Similar phenomena can be observed as the wave length *λ* decreases, and the local back flow becomes prominent when *λ* = 30 Å. Moreover, the back flow mainly happens at the sinusoidal wave crests and troughs where the water molecules need to dramatically change their velocity direction to conform to the morphology of the nanochannel. When the flow velocity is high, the water molecules do not have enough time to change their movement directions and thus collide with GE layers and lead to the local back flow which then disturbs the uniform water flow and cause collision among the water molecules, escalating the water transport resistance.

**Figure 9:**
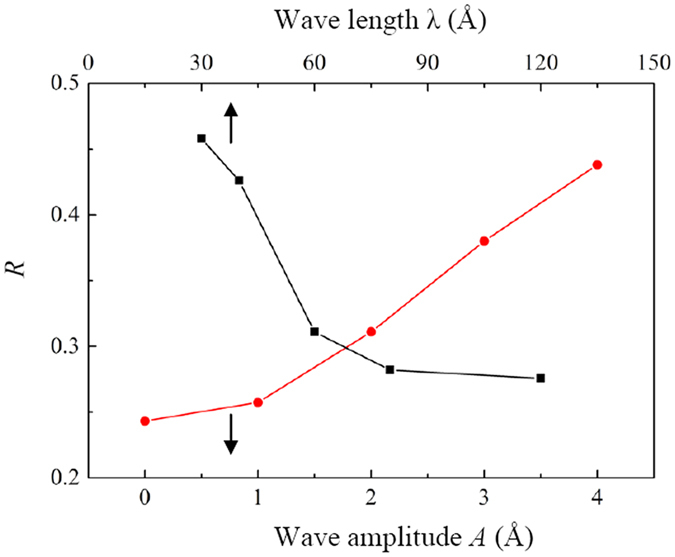
Percentage *R* of the number of water molecules with negative velocity component along the *x*-direction as functions of the wave amplitude *A* and wave length *λ* for *d* = 10 Å.

To quantitively analysis the blocking effect of the GE layers, the percentage *R* of the water molecules with negative velocity component along the *x*-direction is investigated (Fig. 9). Here, *R* is obtained by taking the average value in a period of 50 ps after the steady water flow rate is achieved. It is noted that even for the flat GE layers (*A* = 0 Å), the percentage *R* is still around 0.25, which may be due to the thermal fluctuation and self-diffusion of the water molecules inside the nanochannel. Nevertheless, as the wave amplitude *A* increases or the wave length *λ* decreases, the percentage *R* increases monotonically. At *λ* = 30 and *A* = 4 Å, the percentage *R* is even close to 0.5. This indicates that for a larger curvature of the GE layers, more water molecules are blocked by the channel wall and conflicted with other molecules, leading to a more prominent local back flow effect.

### Distributions of water density, dipole moments and hydrogen bonds

To further understand the molecular mechanism behind the transport resistance induced by the nanochannel morphology, the distributions of the water density, dipole moment orientation and hydrogen bonds are investigated. These distributions are selected for investigation because they well reflect the confinement effect of the GE layer on the water molecule and the interaction between them.

**Figure 10:**
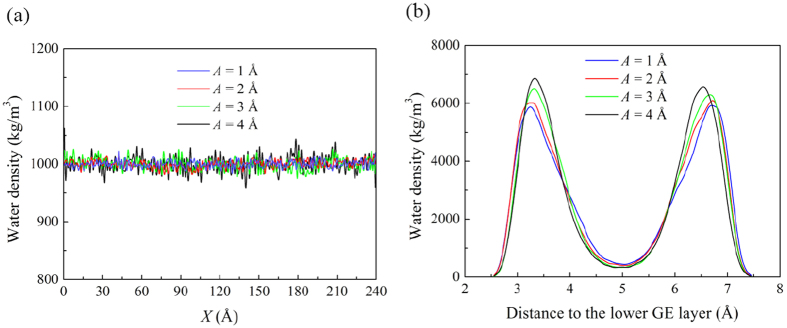
Water density distribution along the (**a**) *x*-direction and (**b**) *z*-direction at various wave amplitudes *A* for the case of *d* = 10 Å, *λ* = 60 Å and phase angle difference *φ* = 0°.

Figure 10 shows both the water density distributions along the *x*- and *z*-directions for the representative case of *d* = 10 Å, *λ* = 60 Å and *φ* = 0°. It can be observed that the water density along the *x*-direction is almost constant. However, the water density distribution along the *z*-direction shows that for all the wave amplitudes *A* investigated, water is significantly densified near the GE layers, forming two first solvation shells (FSSs) near the inner surfaces of the lower and upper GE layers, respectively. This indicates that the layered water structure that forms inside the flat GE bilayers as reported by previous works^25^ is preserved when the morphology of the bilayers changes. As *A* increases, the heights of the two peaks increase slightly and the distance between them also decreases mildly. Similar distribution patterns and changes can be observed for the water density distributions along the *x*- and z- directions for a fixed *A* and a decreasing wave length *λ* (not shown here). These results indicate that as *A* increases or *λ* decreases, the confinement effect of the GE layers on the water molecules becomes stronger, and their accessible volume becomes smaller.

**Figure 11:**
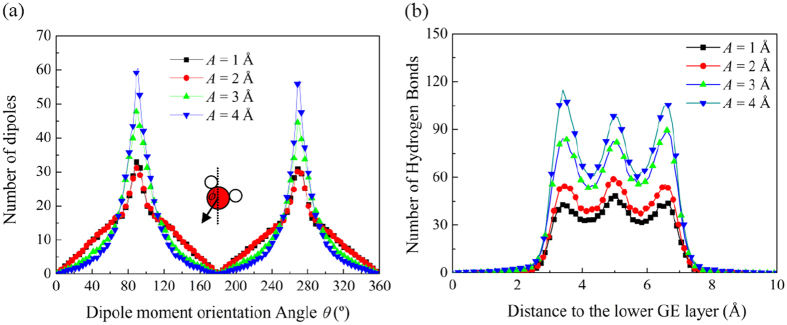
Distributions of (**a**) the dipole moment orientation *θ* and (**b**) the number of hydrogen bonds along the channel thickness *z*-direction at various wave amplitudes *A* for the case of *d* = 10 Å, *λ* = 60 Å and *φ* = 0°.

Due to the reduced accessible volume, the water molecules may need to rearrange themselves to form a more densely packed or ordered structure. Commonly, the degree of ordering of a molecular structure can be characterized by the radial distribution function (RDF) which is calculated based on the distances between the molecules or atoms. The simulation results show that the RDFs for the O-O, O-H and H-H atom pairs do not change significantly when the channel morphology changes. This is mainly because under the strong confinement imposed by the curving GE layers, there is little room left for the water molecules to increase their degree of ordering through rearranging their relative positions. Instead, the simulations show that the water molecules increase their degree of ordering by rotating their dipole moments. Figure 11a shows the distribution of the orientation angle *ϕ* of the water dipole moments in the *x*-*z* plane for various wave amplitudes *A*. Similar results can be obtained for varying the wave length *λ*, and thus are not shown here. It is found that the water molecules tend to prefer *ϕ* = 90° and *ϕ* = 270° to other angles, indicating that they tend to point their OH bond towards the GE layers. Moreover, as the wave amplitude *A* increases, the distribution becomes more concentrated to *ϕ* = 90° and *ϕ* = 270°. As the concentration intensifies, the degree of ordering of the water molecules increases, and thus enhancing the formation of hydrogen bonds between them as discussed below.

Figure 11b plots the hydrogen bond distribution along the channel thickness *z*-direction for various wave amplitudes *A.* Here, the formation of a hydrogen bond is determined by the conditions that (1) the O-O distance of two water molecules is less than 3.5 Å, and (2) the angle between the O-O axis and one O-H bond of these two water molecules is below 30°. The position of the hydrogen bond is determined by the average position of the donor H atom and the acceptor O atom. It is observed that at the same distance to the lower GE layer, a larger wave amplitude *A* always corresponds to a larger number of hydrogen bonds. The hydrogen bonding represents an attractive interaction among the water molecules. As the hydrogen number increases, the interactions enhance, resulting an enhanced water viscosity and thus a larger transport resistance.

Moreover, besides the uneven distribution of the water density, the hydrogen bonds distribution inside the nanochannel is also uneven along the channel thickness direction. There are three peaks which appear at the FSS near the lower GE layer, in the middle plane of the channel and at the FSS near the upper GE layer, respectively. This is mainly caused by the layered structure of the water molecules and the concentration of their dipole moment orientation angles, which makes the formation of hydrogen bonds most easily inside each FSS and between these shells. As the location moves from the FSS to the surface of GE layers, the number of hydrogen bonds decreases sharply, particularly for a large wave amplitudes *A.* This phenomenon is attributed to the fact that most of the water molecules at the FSSs tend to point one of their OH bond to the GE surface and these OH bonds are “free” from the formation of hydrogen bonds. This is consistent with the previous experimental observation that hydrogen bonding between adjacent water molecules at hydrophobic interfaces is weak due to the presence of free OH bonds.

**Figure 12:**
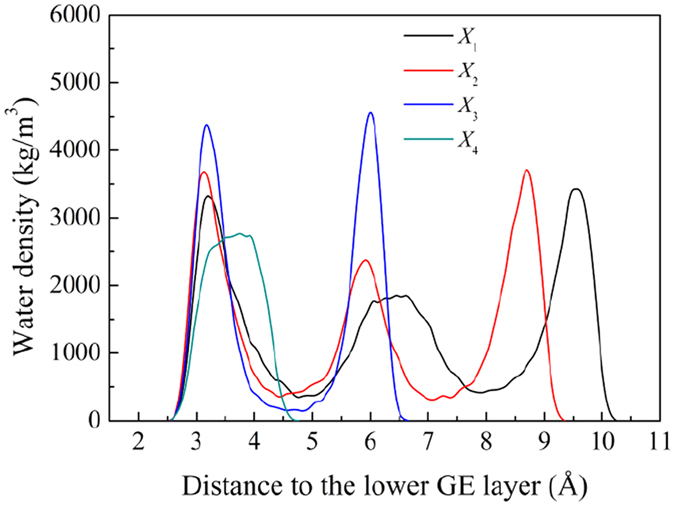
Water density distribution along the *z*-direction at different locations for the phase angle difference *φ* = 90°. In this case, *d* = 10 Å,*λ* = 60 Å and *A* = 2 Å.

Before closing this section, it is worthy to shed some light on the effect of phase difference angle *φ* on the water distribution inside the channel. Taking the case of *φ* = 90° as an example, the water distribution along the *z*-direction for the case of *d* = 10 Å, *λ* = 60 Å and *A* = 2 Å is plotted in Fig. 12. For a non-zero angle *φ*, the distance between the upper and lower GE layers changes as the water molecules move through the nanochannel. Hence, the water distributions along the *z*-direction at different locations along the *x*-direction are plotted. Four locations are chosen with their *x* coordinates as 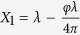, *X*_2_ = *X*_1_ + 5, *X*_3_ = *X*_4_ − 5 and 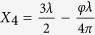. Here, *X*_1_ and *X*_4_ are chosen because they correspond to the maximum and minimum distances between the two GE layers, respectively, according to Eqs. 4 and 5.

It can be seen that as the water molecules move from the location with the maximum interlayer distance to the location with the minimum distance, the water molecules structure changes from three layers to one layer gradually. As a result, during the transportation, the water molecules have to frequently rearrange themselves spatially to conform to the change of the distance between the GE layers. Such rearrangement would cause intensive collisions between the water molecules and also between them and the GE layers, thus disturbing their uniform movement along the flow direction and enhancing the water transport resistance. At the same time, this rearrangement would also lead to frequent break and formation of the hydrogen bonds. As a result, part of the energy imposed by the external pressure is dissipated in this process and the water flow rate is reduced.

When *φ* < 70°, the minimum distance Δ*Z*_min_ between the upper and lower GE layers is larger than 7.70 Å which allows the passing through of two layers of water molecules. When 70° < *φ* < 130°, Δ*Z*_min_ becomes close to and even smaller than 7.0 Å, leading to that only one single layer of water molecules can pass through the channel. This explains the jump of the water deceleration *a*, i.e. the water transport resistance, at *φ* = 70° shown in Fig. 7. When *φ* becomes larger than 130° and gets close to180°, Δ*Z*_min_ approaches to 6.0 Å, a distance below which no water molecules can penetrate through. Correspondingly, a jump occurs at *φ* = 130° for the water deceleration (Fig. 7).

### Potential energy surface

Previously, Yuan *et al*.^32^,^33^ have developed a combined theoretical framework of hydrodynamics and molecular kinetic theory to investigate the substrate morphology effect on the spreading of a droplet on a lyophilic pillar-arrayed surface. It has been demonstrated that as compared with the flow behaviors on a flat surface, those on the rough surface are significantly influenced by the concerted effect of the surface roughness and the liquid-solid interactions. Changes of such interactions would significantly affect the lyophilic property of the solid surface. For the water molecules transport inside the GE bilayers, the interactions between the liquid water molecules and the solid GE walls can be characterized by the potential energy surface (PES). The PES is an important character that could be used to describe the interaction between the liquid water molecules and the solid GE layers for different geometries or surface morphologies. The PES has recently been proven to be critical for the understanding of the dynamics and transport behaviors at the atomic scales^15^,^34^,^35^.

Based on the PES, the activation energy for a water molecule moving from one location to another can be calculated, providing a direct explanation for the origin of water transport resistance inside the GE bilayers. In this work, the PES is obtained by using one water molecule as a probe to screen the surface of the GE layers. During the screening process, the potential energy of the system is recorded as the probe water molecule moves. The distance of the probe water molecule to the lower GE layer is set as 3.2 Å or 5.0 Å, which corresponds to two unique surfaces, respectively, i.e., the FSS formed by the water molecules (Fig. 10b) and the middle plane surface (MPS) of the nanochannel.

**Figure 13:**
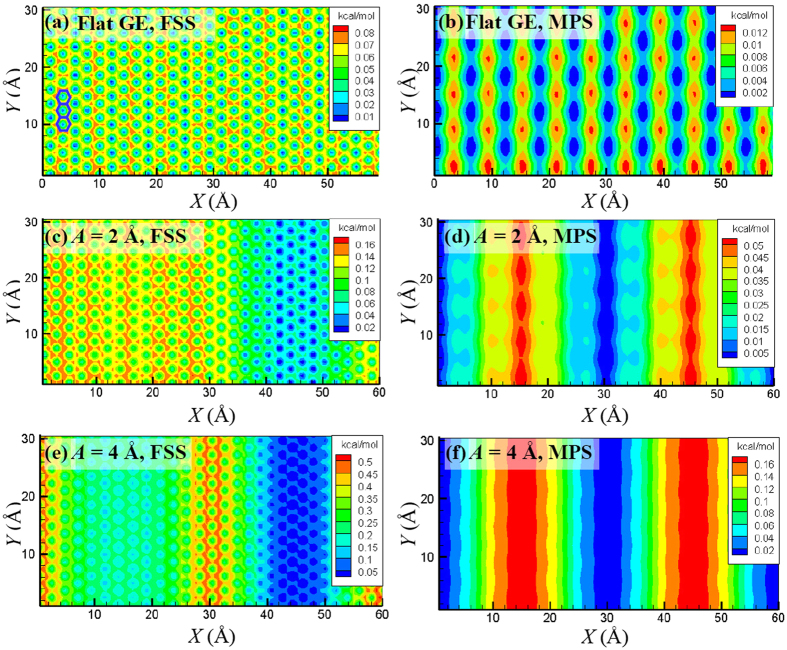
PESs along (**a,c,e**) the FSS and (**b,d,f**) the MPS with various wave amplitudes *A* for *d* = 10 Å, *λ* = 60 Å and *φ* = 0°. In (**a**), three hexagonal rings in blue are drawn to show the mapping between the PES profile and the honeycomb lattice structure of GE.

Figure 13 exemplifies the PESs along the FSS and MPS within a wave length for various wave amplitude *A* with *d* = 10 Å, *λ* = 60 Å and *φ* = 0°. For the sake of comparison and clarity, the minimum potential energy is shifted to zero for each PES. It is found that the profile of the PES highly depends on its distance to the GE layer and also its surface morphology. For a flat GE layer, the PES at the FSS maps well with the honeycomb lattice structure of GE. The minimum potential energy is located around the hollow site of the honeycomb lattice structure while the maximum energy is located around the site on top of the C atoms, which agrees well with previous works^25^,^35^. The PES at the MPS shows a dramatically different pattern as compared to that at the FSS, though both are highly localized along both the *x*- and y-directions with a periodic and alternative pattern.

When the GE layers become asperous, the PES at the FSS is significantly affected by the change of the morphology of GE layers. The mapping between the honeycomb lattice structure of GE and the PES can still be observed. However, the potential energy near the wave nodes tends to have the highest value. Interestingly, the potential energies at the locations near the wave crests are slightly higher than those near the wave troughs. Similar phenomena can be observed when the wave length *λ* decreases with a fixed wave amplitude *A*.

For the curving GE layers, the PES at the MPS tends to be even along the *y*-direction and only changes alternatively along the *x*-direction. There is no longer mapping between the honeycomb lattice structure of GE and the PES profile. In contrast to the PES at the FSS, the minimum potential energy for the PES at the MPS locates at the wave nodes while the maximum locates at the wave crests and wave valleys. In addition, the asymmetry of the PES about the wave nodes as appears in the PES at the FSS disappears. Moreover, as the wave amplitude *A* increases, the widths of the regions with maximum or minimum potential energy increase significantly.

**Figure 14:**
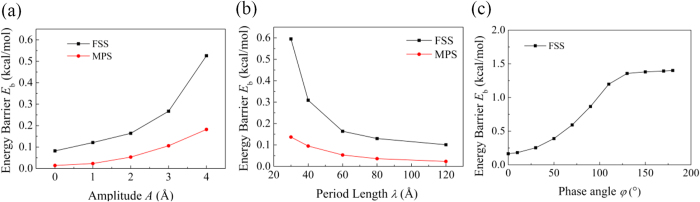
Dependence of the energy barrier *E*_b_ on (**a**) wave amplitude *A*, (**b**) wave length *λ* and (**c**) phase angle *φ* for the interlayer distance *d* = 10 Å. In (**a**), *λ* = 60 Å, *φ* = 0°; in (**b**), *A* = 2 Å, *φ* = 0°; in (**c**) *λ* = 60 Å, *A* = 2 Å.

An important characteristic that can be observed from the PES is that the energy difference between the maximum and minimum potential energies increases as the wave amplitude *A* increases. Here, the energy difference is denoted as the energy barrier *E*_b_ for water molecules to transport through the nanochannel formed by the GE layers. Figure 14a plots the dependence of *E*_b_ on *A* for water molecules move along the FSS or the MPS. It is found that both the *E*_b_ for the FSS and MPS increase monotonically with *A*. Moreover, *E*_b_ for the MPS is much smaller than that for the FSS due to the larger distance between the MPS and the GE layer. This explains that the water flow velocity for a large channel thickness *d* is larger than that for a small *d*, since the average distance of the water molecules to the GE layers increases as *d* increases, and thus leading to a smaller energy barrier for transport.

The dependences of the energy barrier *E*_b_ on the wave length *λ* and the phase angle *φ* are also plotted (Fig. 14b and c). As *λ* decreases or *φ* increases, *E*_b_ increases monotonically. Moreover, as *λ* decreases, the water molecules would need to overcome the energy barrier more frequently as they transport through the channel. With the increased energy barriers, the increase in the transport resistance and thus the reduction in the water flow velocity can be expected according to the recently developed extended Frenkel-Eyring (FE) molecular kinetic model by Wang *et al*.^36^. This model describes the boundary slip behaviors of the fluid in between two confining walls with textured surfaces. The textured surfaces investigated in this model have similar periodic morphologies with the GE bilayers studied in this work.

In the extended FE model, the potential energy barrier *E*_b_ for the movement of the water molecules on the textured surface is demonstrated to be the dominant factor that affects the boundary slip velocity *V*_s_ of the fluid on the texture surface. Their relation can be described as


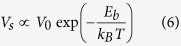


where *V*_0_ = 2*lk*_B_*T*/*h*, *k*_B_ is the Boltzmann constant, *T* is the absolute temperature, and *h* is the Planck constant, *l* is the equilibrium separation between the liquid molecules. According to this model, the boundary slip velocity decreases exponentially with the potential energy barrier. For the water molecules transport inside the GE bilayer, they are highly confined by the GE layers and the boundary slip velocity plays a significant role in affecting the overall transport velocity. Hence, for a large curvature of the GE layers, the large potential energy barrier leads to the low water flow velocity. It is noted that in the extended FE model, the potential energy barrier is averaged over the entire textured surface area. In the current work, the potential energy barrier is defined as the energy difference between the sites with the highest and lowest potential energy. Though the definitions of the energy barrier are different, the extended FE model can still be used to correlate the PES with the water flow velocity in the GE bilayers.

## Conclusions

The channel morphology effect on the water transport behaviors inside the GE bilayers has been studied via MD simulations. The simulation results show that under the same driving force, the water flow velocity decreases as the curvature of the GE layers increases particularly when they corrugate in a non-synergic pattern. For a larger interlayer distance, the same trends can be observed, but the water flow velocity becomes larger. The transport resistance of the water molecules inside the nanochannel of GE layers has been characterized with the deceleration of water molecules when they are allowed to flow freely inside the channel. A close correlation has been found between the water flow velocity and the transport resistance. The transport resistance for water flow along the wave length direction of sinusoidal GE layers is found to be larger than those for other flow directions. The blocking effect of the GE layers is found to be responsible for the resistance, and this blocking effect enhances as the wave amplitude increases or wave length decreases, and even leads to local back flows.

To understand the water transport resistance for different nanochannel morphologies, the distributions of water density, dipole moment orientations and hydrogen bonds inside the channel are investigated. It has been found that the water molecules show layered structure along the channel thickness direction. When the upper and lower GE layers corrugate in a non-synergic pattern, the number of water molecule layers changes as the water molecules move from the location with maximum interlayer distance to the location with the minimum distance. As a result, the water molecules need to frequently rearrange themselves spatially to conform to the morphology the GE layers. Such rearrangement would cause intensive collisions among the water molecules and also between them and the GE layers, leading to increased transport resistance. Moreover, the orientation of water dipole moment tends to prefer 90° and 270° to other angles, indicating that the water molecules tend to point their OH bond to the GE layers. As the wave amplitude increases and wave length decreases, the orientation distribution of the dipole moments becomes more concentrated to 90° and 270°, leading to the increase of the degree of ordering of the water molecules and enhancing the formation of hydrogen bonds between them.

The potential energy surfaces (PESs) with different distances to the lower GE layer have also been analyzed. The results show that the morphology of the GE layers has great impacts on the PES profile. The profiles of PES along the first solvation shell (FSS) show good mapping with the honeycomb lattice structure of GE. However, such mapping is absent for the PES along the middle plane surface (MPS). For the PESs along the FSS, the highest potential energy occurs at the sinusoidal wave nodes, but it occurs at the wave crests and valleys for the PESs along the MPS. The energy barrier for a water molecule to transport through the nanochannel of the GE bilayers increases monotonically as the wave amplitude *A* increases, wave length *λ* decreases or the phase angle difference *φ* increases, which explains the dependences of the water flow velocity and transport resistance on the channel morphology.

The results obtained in the present study are helpful for the understanding of water transport in nanoconfiments particularly with the effect of morphology of the confining walls being taken into account. As the potential value of multilayer GE-based membranes in the applications of nanofiltration is being more and more recognized, this study is of significance to provide guidance for the design and application of such GE-based membranes in future nanofiltration and water purification technologies.

## Methods

According to the experimental findings of the roughness of GE nanosheets^37^,^38^, the wave amplitude *A* is taken to be less than 5 Å. Moreover, the ratio of *A*/*λ* is kept to be smaller than 0.1 to avoid large distortion and strain of the lattice structure of GE. The length *L* of the GE sheets is set to be around 240 Å which is several times of the wave length *λ*, and the width *w* is set to be around 50 Å. Periodic boundary conditions are applied along both the *x*- and *y*-directions. The density of water molecules inside the accessible volume of the nanochannel is set to that of bulk water ~1 g/cm^3^. Here, the accessible volume to the water molecules is deduced from the effective thickness of the channel which is estimated as *d*_eff_ = *d* − 2*σ*_c_ with *σ*_c_ being the commonly used van der Waals radius 1.7 Å of the C atoms^13^,^39^.

All the simulations are conducted by using the large-scale atomic/molecular massively parallel simulator (LAMMPS) package^40^. The Transferable Intermolecular Potential 3 P (TIP3P) water model^41^ is adopted to construct the water molecules with the partial charges of the O and H atoms being set to be −0.834e and 0.417e, respectively. The CHARMM27 force field^42^ is used to describe both the bonded and non-bonded interatomic interactions between the O and H atoms. The long-range electrostatic interactions are computed with the particle-particle particle-mesh (PPPM) method with a cutoff distance of 12 Å and a root mean square accuracy of 10^−4^. The interaction between the GE bilayer and water molecules is modeled as vdW interactions between the C and O atoms, while the interaction among the water molecules is modeled as vdW interactions of the H-O, H-H and O-O atom pairs.

In this study, all the vdW interactions are described by the 12-6 Lennard-Jones potential as


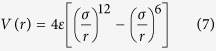


Here, *r* represents the distance of two atoms, *ε* is the energy well that reflects their interaction strength, and *σ* denotes the zero-across distance of the potential. The values of *σ* and *ε* for the pair interactions of H-O, H-H and O-O are adopted as 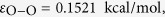 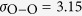 Å, 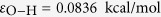, 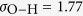 Å, 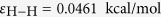 and 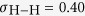 Å, according to the CHARMM27 force field^42^, while those for the C-O interaction are 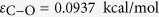 and 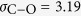 Å. This C-O interaction parameter set has previously proved to be capable of well reproducing the experimentally observed contact angle of water droplet on a GE nanosheet^43^. The cutoff distance for the vdW interactions is set as 10 Å, which is about three times *σ*_C−O_ = 3.19 Å.

The transport behavior of liquids in a confining nanochannel is a non-equilibrium process, and the non-equilibrium MD simulation is usually employed during which the flow is driven by an external pressure for computational simplicity. In this study, the effect of pressure is realized by directly applying a constant force of 2.5 × 10^−3^ kcal/mol/Å along the *x*-direction to the oxygen atom of each water molecules. This corresponds to a pressure difference of ~580 MPa across the two ends of the GE bilayers. As the water flows through the nanochannel between the GE layers, these layers are treated as rigid walls and their flexibilities are not considered although they can affect the free energy of water filling and emptying the nanochannel^44^. In the present model, the water molecules are filled inside the nanochannel artificially at the initial stage of the simulation. In addition, the effect of nanosheet flexibility is significant only for self-diffusion at a low pressure loading. For the pressure applied in the present model, such effect is negligible. Furthermore, the GE layers would also deviate from the initially designed sinusoidal profile if they are allowed to move flexibly, which makes it difficult to quantitatively correlate the morphology of the GE sheets with the water transport behaviors.

During the simulations, the constant volume and temperature (NVT) ensemble is adopted to conserve the system temperature at 300 K using the Nosé-hoover thermostat with a damping parameter of 0.05 ps^−1^. The temperature is calculated with the center-of-mass velocity subtracted. Such thermostat coupling strategy was adopted in many previous studies concerning the transport behaviors of water molecules in carbon nanotubes^1^,^30^,^31^ and GE nanosheets^14^,^34^. It has been demonstrated that a direct coupling of the fluid confined in a nanochannel with a thermostat might perturb the dynamic behaviors of the fluid, and instead the coupling of the confining walls of the fluid with the thermostat to control the temperature and to dissipate the energy imposed by the external pressure would be better and also closer to practical experimental set-ups^45^. It has also been reported that such perturbation would become prominent only when a non-slip and strongly sheared condition is assumed at the fluid-solid boundary^46^. For a weakly sheared and slip boundary condition which holds for the water flow inside the GE layers, both the above mentioned two thermostat coupling strategies will produce similar behaviors.

Under the NVT ensemble, the water molecules are initially relaxed for 50 ps with a timestep of 0.5 fs to reach an equilibrium state. Afterwards, the driving force is imposed to generate a flow through the nanochannel. With the driving force imposed, the simulation is conducted for 250 ps to obtain a steady water flow. Upon reaching the steady state, the simulation is conducted for another 250 ps for data collection and statistical averaging.

## Additional Information

**How to cite this article**: Liu, B. *et al*. Channel morphology effect on water transport through graphene bilayers. *Sci. Rep.* **6**, 38583; doi: 10.1038/srep38583 (2016).

**Publisher’s note:** Springer Nature remains neutral with regard to jurisdictional claims in published maps and institutional affiliations.
